# Outcomes in ST-segment elevation myocardial infarction treated with primary percutaneous coronary intervention or pharmacoinvasive strategy in a Latin American country

**DOI:** 10.1186/s12872-022-02730-6

**Published:** 2022-06-29

**Authors:** Manuel Chacón-Diaz, Piero Custodio-Sánchez, Paol Rojas De la Cuba, Germán Yábar-Galindo, René Rodríguez-Olivares, David Miranda-Noé, Luis Marcos López-Rojas, Akram Hernández-Vásquez

**Affiliations:** 1 Instituto Nacional Cardiovascular INCOR, Essalud, Lima, Peru; 2 Universidad Científica del Sur, Lima, Peru; 3 Hospital Nacional Almanzor Aguinaga Asenjo, Essalud, Chiclayo, Peru; 4 Hospital Nacional Guillermo Almenara, Essalud, Lima, Peru; 5 Hospital Nacional Alberto Sabogal, Essalud, Callao, Peru; 6 Centro de Excelencia en Investigaciones Económicas y Sociales en Salud, Vicerrectorado de Investigación, Universidad San Ignacio de Loyola, Lima, Peru

**Keywords:** Myocardial infarction, Reperfusion, Mortality, Heart failure, Peru

## Abstract

**Objective:**

The primary percutaneous coronary intervention (PPCI) is the preferred reperfusion strategy for ST-segment elevation myocardial infarction (STEMI). The pharmacoinvasive strategy (PIs) is a reasonable alternative when prompt PPCI is not possible, especially in resource-limited regions. We aimed to compare PPCI versus PIs outcomes in Peru.

**Methods:**

This was a retrospective cohort study based on the second Peruvian Registry of STEMI (PERSTEMI II). We compared the characteristics, in-hospital outcomes and 30-day mortality of patients undergoing PPCI during the first 12 h and those receiving a PIs. A propensity score-matched analysis was conducted to compare the effects of each treatment strategy on clinical outcomes.

**Results:**

PIs patients were younger than PPCI patients, had a shorter first medical contact time, first medical contact to reperfusion time, and total ischemic time until reperfusion. Successful PCI was more frequent in the PIs group (84.4% vs. 71.1%, *p* = 0.035). There were no differences between PIs and PPCI in terms of total in-hospital mortality (5.2% vs. 6.6%, *p* = 0.703), cardiovascular mortality (4.2% vs. 5.3%, *p* = 0.735), cardiogenic shock (8.3% vs. 13.2%, *p* = 0.326), heart failure (19.8% vs. 30.3%, *p* = 0.112), or major bleeding (0% vs. 2.6%, *p* = 0.194). In the propensity score-matched analysis, the rates of cardiovascular mortality, postinfarction heart failure and successful reperfusion were similar.

**Conclusions:**

In this real-world study, no differences were found in the in-hospital outcomes between patients with STEMI who received PIs or PPCI.

## Background

ST-segment elevation myocardial infarction (STEMI) requires timely reperfusion therapy which may be carried out by two treatment strategies: primary percutaneous coronary intervention (PPCI) [1] or a pharmacoinvasive strategy (PIs). A lower mortality rate has been reported in patients who receive PPCI in clinical trials conducted in high-volume centers and with adequate ischemic times [2]. Within the PIs, which is defined as the early administration of fibrinolytic therapy with a subsequent percutaneous coronary intervention (PCI), an early systematic percutaneous coronary intervention performed after successful fibrinolysis is an acceptable strategy to reduce cardiovascular events [3].

The choice of reperfusion strategy depends on several factors. In the case of PPCI, adequate hospital infrastructure along with suitable equipment and logistics are required, in addition to trained human resources [1]. Another relevant factor to be considered is the delay in time until the selected strategy is applied. It is recommended that reperfusion be performed within the first 12 h after symptom onset, since the earlier the therapy is applied the higher the survival rate. However, in a large percentage of patients, PPCI is not achieved within the recommended time and is associated with an increase in morbidity and mortality [3, 4]. This increase in morbidity and mortality has been described in international registries, reporting worse outcomes in the 5-year follow-up of patients undergoing late PPCI compared to patients undergoing a PIs [5].

In low-to-middle-income countries, the proportion of patients with STEMI who receive a prompt PPCI reperfusion is low. Therefore, the greater availability and relative simplicity associated with the administration of a fibrinolytic agent with the PIs makes this approach a reasonable alternative when prompt PPCI (within the first 120 min of diagnosis) cannot be administered [1]. Taking into account the fragmented health care system of Peru, in addition to the difficulty in accessing health services and lack of coronary intervention capable centers, one-third of the population in the PERSTEMI II registry did not receive any reperfusion therapy, and 26% of patients received fibrinolysis followed by PCI [6]. Furthermore, in our region no study has compared the in-hospital outcomes between PI and PPCI strategies in a real-world setting. Therefore, the objective of our study was to compare in-hospital outcomes and 30-day mortality of STEMI patients treated by a PIs or PPCI in a cohort of patients included in the second Peruvian registry of STEMI (PERSTEMI II).

## Methods

### Design

The PERSTEMI II registry [6] is a previously published, prospective, multicenter study that analyzed the clinical characteristics and outcomes of patients with a diagnosis of STEMI treated in level III public hospitals in the main cities of Peru during 2020. In this substudy, two reperfusion strategies, PPCI and PIs, were compared. The factors considered included the characteristics of STEMI presentation, delay in treatment and in-hospital outcomes, among others.

The PPCI group included patients with STEMI who underwent coronary angiography, primary angioplasty, and intracoronary stent placement as the first reperfusion therapy within the first 12 h of symptom onset. The PIs group included patients with STEMI who received fibrinolysis as the first reperfusion therapy within the first 12 h of symptom onset followed by routine coronary angiography and coronary angioplasty within 3–24 h after fibrinolytic agent administration in the case of successful fibrinolysis and immediately in the case of failed fibrinolysis. Patients receiving no reperfusion treatment during the first 12 h of evolution, those with fibrinolysis alone, and patients receiving no reperfusion at any time were excluded from the analysis.

### Study variables

The variables studied were age, sex, medical history, and cardiovascular risk factors (arterial hypertension, diabetes mellitus, dyslipidemia, smoking habit, chronic kidney disease, previous myocardial infarction, previous coronary revascularization, and cerebrovascular disease). Furthermore, we evaluated the Killip–Kimball classification on admission, time until the first medical contact, time from symptom onset to reperfusion, PCI characteristics, complications, and success of reperfusion (thrombolysis in myocardial infarction [TIMI] 3 flow after PCI). In-hospital mortality, cardiovascular mortality (due to cardiac arrest or cardiogenic shock), post infarction heart failure (symptoms and signs of heart failure during hospitalization), mechanical complications (presence of septal intraventricular rupture or free wall rupture or papillary muscle rupture during hospitalization), mayor bleeding (intracerebral hemorrhage or > 5 mg/dL reduction in hemoglobin level) and cerebrovascular events (ischemic or hemorrhagic stroke during hospitalization) were also evaluated.

The endpoints evaluated were the composite of in-hospital death and post infarction heart failure, and the secondary endpoint was reperfusion success (TIMI 3 flow in the coronary artery related to infarction, after stent placement) of both strategies.

### Statistical analysis

Categorical variables were expressed in frequencies and percentages and numerical variables were expressed as means or medians with their respective measures of dispersion according to the Shapiro–Wilk test. The association between categorical variables was assessed using the chi-square test, and the relationship between numerical variables was evaluated using the Student’s *t* test (normal distribution) or Wilcoxon rank sum test (nonparametric distribution). The survival rate by reperfusion strategy was estimated by Kaplan–Meier analyses.

A propensity score analysis was performed to yield a balanced distribution of covariates and to estimate the PIs effects on the primary outcome (30-day cardiovascular death or symptomatic heart failure) and secondary outcome (successful reperfusion after PCI) of both. We calculated the propensity score using a multivariable logistic regression model, in which the treatment exposure (PIs) was regressed as a dependent variable for relevant covariates. The following covariates of clinical and epidemiological relevance were included to establish the propensity score: age; sex; history of arterial hypertension, diabetes mellitus, and chronic kidney disease; cardiac arrest on admission; TIMI flow pre-PCI, total ischemic time to reperfusion, time to first medical contact and localization of the infarction. Thereafter, kernel matching with a bandwidth of 0.06 was performed with the command *psmatch2*. All statistical analyses were performed using the STATA 14.0 program. The significance level was set at p < 0.05.

### Ethics

The study was conducted after obtaining approval of the Ethics and Research Committee of the National Cardiovascular Institute, INCOR.

## Results

**Fig. 1:**
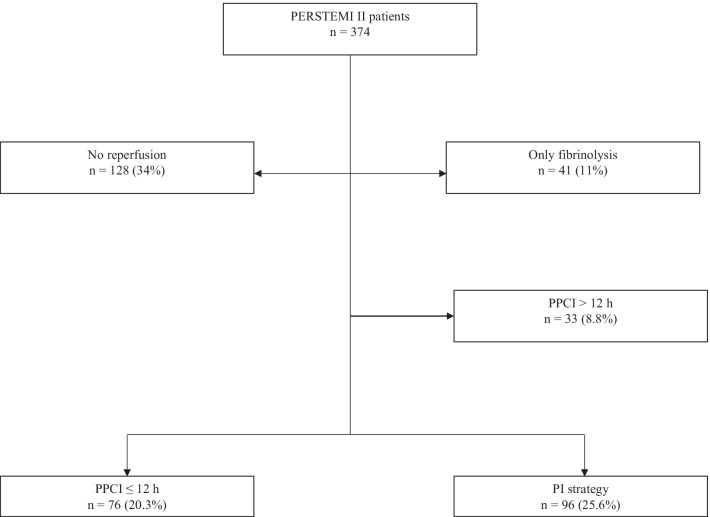
Flow chart representing participant selection according to reperfusion strategies in the PERSTEMI II registry. *PPCI* primary percutaneous coronary intervention, *PI* pharmacoinvasive strategy

The PERSTEMI II registry included 374 patients with STEMI. After applying the exclusion criteria, we found 76 patients who received PPCI within ≤ 12 h and 96 underwent the PIs, comprising the final study sample (Fig. 1). In the PIs group, fibrinolysis was unsuccessful in 27 patients (28%) and they were treated with rescue angioplasty (15.7% of all procedures).

The median age was lower in the PIs group than in the PPCI group (62 years (interquartile range [IQR]: 52–70) vs. 68 years, IQR: 59–75, *p* < 0.001). The predominant sex was male, and the main antecedents were arterial hypertension and dyslipidemia (Table 1). Additionally, typical angina, dyspnea, anterior localization, and Killip–Kimball I hemodynamic status on admission were the most frequent presentations in both groups, with no significant differences between them (Table 2).

**Table 1:** Epidemiological findings of the study groups

Characteristic	PIs group n (%)	PPCI group n (%)	*p*
Age (in years) (median, IQR)	62 (52–70)	68 (59–75)	< 0.001
Male sex	84 (87.5)	70 (92.1)	0.453
Clinical Characteristics			
Hypertension	42 (43.7)	37 (48.7)	0.519
Type 2 diabetes mellitus	26 (27.1)	14 (18.4)	0.182
Dyslipidemia	42 (43.8)	39 (48.6)	0.519
Smoking habit	21 (21.9)	28 (36.8)	0.031
Chronic coronary syndrome	4 (4.2)	3 (3.9)	1.000
Cerebrovascular disease	6 (6.3)	2 (2.6)	0.469
Previous myocardial infarction	4 (4.2)	3 (3.9)	0.942
Coronary revascularization	2 (2.1)	3 (3.9)	0.656
Chronic kidney disease	5 (5.2)	5 (6.6)	0.751
Chronic heart failure	0	1 (1.3)	0.442

Categorical values are expressed in frequency and percentage (%)

*IQR* interquartile range, *PIs* pharmacoinvasive strategy, *PPCI* primary percutaneous coronary intervention

*P* value obtained using Pearson’s chi-square and Wilcoxon rank sum test according to the type of variables

**Table 2:** Myocardial infarction presentation according to study group

Characteristic	PIs group n (%)	PPCI group n (%)	*p*
Symptoms			
Typical angina	95 (98.9)	72 (94.7)	0.102
Atypical chest pain	1 (1.04)	3 (3.9)	0.322
Dyspnea	18 (18.8)	15 (19.7)	0.870
Syncope	3 (3.1)	3 (3.9)	1.000
Cardiac arrest	1 (1.04)	4 (5.2)	0.102
Electrocardiogram on admission			
Atrial fibrillation	3 (3.1)	1 (1.3)	0.06
High-grade atrioventricular block	1 (1.1)	6 (7.9)	0.06
Localization of infarction			
Anterior	63 (65.6)	43 (56.6)	0.374
Inferior	34 (33.0)	33 (43.4)	0.441
Lateral	1 (1.4)	–	0.295
KK classification on admission			
KK I	68 (70.8)	49 (64.5)	0.211
KK IV	3 (3.1)	4 (5.3)	0.390

*KK* Killip–Kimball, *PPCI* primary percutaneous coronary intervention, *PIs* pharmacoinvasive strategy

Patients in the PIs group received full dose alteplase according to weight, IV enoxaparin, aspirin 325 mg and clopidogrel 300 mg as antiplatelet drugs (91.5%), and 8.5% of the cases received ticagrelor as a second antiplatelet agent. In the PPCI group patients received aspirin 325 mg and clopidogrel 300 mg loading dose plus IV enoxaparin at 1 mg/kg, and 7% of PCI cases received ticagrelor as a second antiplatelet agent. GPIIb/IIIa inhibitors were not used because they are not available in our country.

### Delays in reperfusion

**Fig. 2:**
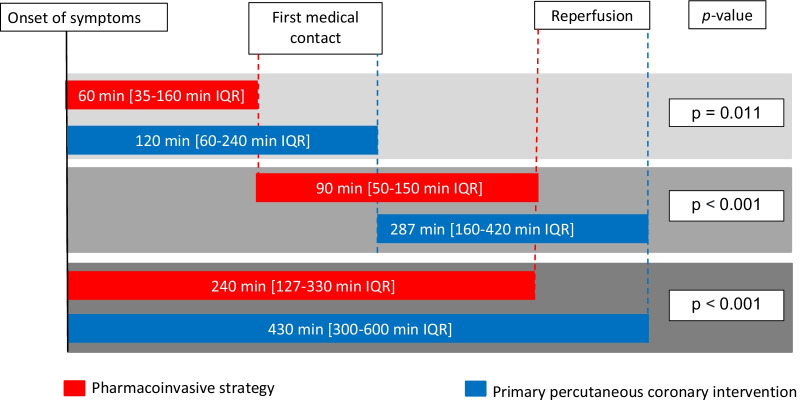
Time delays until the first medical contact and reperfusion according to the study groups. *IQR* interquartile range

The median time from symptom onset to the first medical contact (FMC) was 60 min (IQR: 35–160 min) in the PIs group and 120 min (IQR: 60–240 min) in the PPCI group (p = 0.010). The time from FMC to reperfusion was 90 min (IQR: 50–150 min) in the PI group and 287 min (IQR: 160–420 min) in the PPCI group (p < 0.001). The total ischemic time (from symptom onset to reperfusion) was 240 min (IQR: 127–330 min) in the PI group and 430 min (IQR: 300–600 min) in the PPCI group ﻿(p < 0.001) (Fig. 2). In cases of fibrinolysis failure, PCI was immediately performed within a median time of 90 min (IQR: 30–270).

One hundred thirty-five patients (78.5%) had a FMC within less than 3 h of symptom onset. 58.5% underwent a PIs and 41.5% the PPCI. Among patients having FMC within 3 to 12 h of symptom onset, 45.9% underwent a PIs and 54.1% PPCI (*p* = 0.172).

### Characteristics of coronary angiography

The most affected infarct-related artery (IRA) in the PIs and PPCI groups was the anterior descending artery (65.6 and 56.6%, respectively), followed by the right coronary artery (29% and 37%, respectively), and to a lesser extent, the circumflex artery (*p* = 0.459). Coronary stents were placed in 89 PIs group patients (92.7%) and 70 PPCI group patients (92.1%) *(p* = 0.882). In the PIs group, seven patients did not undergo stent placement due to the presence of unfavorable coronary anatomy. Six patients in the PPCI group did not undergo stent placement due to unsuccessful angioplasty (four patients), mechanical complication (one patient), or the diagnosis of myocardial infarction with non-obstructive coronary arteries (one patient).

Furthermore, 12.5% of cases in the PIs group and 55.3% cases in the PPCI group presented with occluded IRA at the time of initial coronary angiography. The success rate of mechanical reperfusion (final TIMI 3 flow) was 84.4% and 71.1% in the PIs and PPCI groups, respectively (*p* = 0.035). Multivessel disease was found in 52.1% of cases in the PIs group and 53.9% of cases in the PPCI group (*p* = 0.808). Angioplasty of non-IRA was more frequent in the PIs than in the PPCI group (74.5% vs. 54.7%, *p* = 0.046), and this was mainly performed during the initial intervention [39.5% (PIs group) vs. 8.7% (PPCI group), *p* = 0.017].

Radial access was performed in 95.4% of patients, while femoral access was carried out in 8 patients (2 in the PIs and 6 in the PPCI group).

### Mortality and in-hospital adverse events

There were five (5.2%) in-hospital all-cause deaths in the PIs group and five (6.7%) in the PPCI group (relative risk [RR]: 0.79, 95% confidence interval [CI]: 0.23–2.63, *p* = 0.702]. Cardiovascular mortality was 4.2% in the PIs group and 5.3% in the PPCI group (*p* = 0.735).

There were no differences of postinfarction heart failure, major bleeding, or other in-hospital complications rates between the two groups (Table 3), neither in the hospital stay (median: 6 days, IQR: 5–11 in the PI group; median: 7 days, IQR: 5–11 in the PPCI group; *p* = 0.414).

**Table 3:** Mortality and in-hospital outcomes according to the study groups

In-hospital outcomes	PIs group n (%)	PPCI group n (%)	*P*
All cause mortality	5 (5.2)	5 (6.6)	0.703
Cardiovascular mortality	4 (4.2)	4 (5.3)	0.735
Postinfarction heart failure	19 (19.8)	23 (30.3)	0.112
Cardiogenic shock	9 (8.3)	10 (13.2)	0.326
Postinfarction angina	5 (5.2)	3 (3.9)	1.000
Mechanical complication of infarction*	1 (1.04)	3 (3.9)	0.322
Cerebrovascular event	1 (1.04)	0	-
Major bleeding	0	2 (2.6)	0.194

*PIs* pharmacoinvasive strategy, *PPCI* primary percutaneous coronary intervention

*Interventricular septal or free wall rupture

**Fig. 3:**
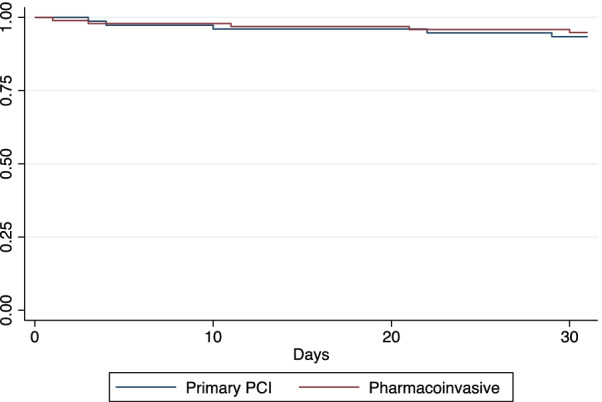
Kaplan–Meier survival rate by reperfusion strategy

At 30 days, cardiovascular mortality remained unchanged compared to in-hospital cardiovascular mortality, only one patient died after discharge and before 30 days due to infectious disease. The Kaplan–Meier survival curve showed no differences in 30-day survival between the two groups (Fig. 3).

### Effects of PIs on the events of interest

Table 4 shows the distribution and comparison of covariates between the exposed and unexposed groups in matched and unmatched samples. The matching resulted in a total of 85 PIs patients and 73 PPCI patients.

**Table 4:** Comparison of background covariates between exposure groups in the unmatched and matched samples

Variable	Unmatched (U)	Mean		% bias
	Matched (M)	Treated	Control	
Male patient	U	0.88	0.92	− 15.2
	M	0.91	0.86	15.3
Age in years	U	61.12	67.08	− 51.6
	M	62.06	60.80	10.9
Anterior/septal/lateral localization	U	0.66	0.57	18.5
	M	0.64	0.63	1.2
Initial TIMI	U	0.45	0.11	82.5
	M	0.39	0.50	− 27.4
Total ischemia-to-reperfusion time	U	259.41	444.66	− 100
	M	258.12	314.55	− 30.5
Time to first contact	U	115.45	161.87	− 36.3
	M	118.74	117.21	1.2
Hypertension	U	0.44	0.49	− 9.9
	M	0.45	0.63	− 37.4
Diabetes mellitus	U	0.27	0.18	20.7
	M	0.25	0.39	− 33.7
Chronic kidney disease	U	0.05	0.07	− 5.8
	M	0.06	0.09	− 12
Cardiac arrest	U	0.06	0.08	− 6.4
	M	0.07	0.02	19.6

The estimate of the PIs effect is shown in Table 5. The table shows the average treatment effects on the treated (ATT) for the outcomes. No differences were found between the effect of PIs and PPCI on 30-day cardiovascular death or symptomatic heart failure. There was also no difference between PIs and PPCI on successful mechanical reperfusion rate.

**Table 5:** Estimated average treatment effects on the treated according to coronary intervention of the two groups (*PPCI* primary percutaneous coronary intervention, *PIs*: pharmacoinvasive strategy) adjusted for covariates

Outcome variable	Sample	Treated	Control	Difference	S.E	P value
30-day cardiovascular death or symptomatic heart failure	ATT	0.212	0.295	− 0.083	0.132	0.527
Successful reperfusion after PCI	ATT	0.824	0.922	− 0.099	0.065	0.127

Covariates include: age, sex, initial TIMI, total ischemia-to-reperfusion time, time to first contact, hypertension, diabetes mellitus, chronic kidney disease, cardiac arrest, and location)

*ATT* average treatment effects on the treated; S.E. definer, *PCI* percutaneous coronary intervention

## Discussion

In this substudy of the PERSTEMI II registry, we did not find any differences in outcomes such as in-hospital mortality or heart failure between STEMI patients treated with PPCI or PIs. This can possibly be attributed to the short time between ischemia onset and reperfusion observed in the PIs group.

This is the first registry on this subject in Peru, in which there is a high percentage of reperfusion failure due to inaccessibility to hemodynamic rooms and shows the application of fibrinolysis alone as the only one reperfusion strategy in many cases. Our records evidenced that fibrinolysis is the most frequently used initial reperfusion strategy in Peru (37% of cases), similar to the 38% reported in the PERSTEMI I study [7] in Peru and in the *Tercer Registro Nacional de Síndromes Coronarios Agudos* (RENASICA III) [8] in Mexico, and is higher than the 22% reported in the *Registro Nacional de Infarto de Miocardio con elevación del Segmento ST* (ARGEN-IAM-ST) [9] study in Argentina.

Furthermore, more than 50% of the patients who achieved successful fibrinolysis underwent coronary angiography within the next 24 h following the PIs. This value is similar to the 48.5% value reported in the PERSTEMI I study [7] conducted in 2017, and showed no significant variations in transfer rates for completing the PIs despite the current pandemic situation.

As this was an observational study, the indication for post-fibrinolysis PCI was not in the hands of the research team, but rather depended on the treating physician. The main reason why PCI was not performed after fibrinolysis in 55.8% of patients was the lack of hemodynamic rooms to perform the procedure because the patients were treated in the provinces and most belonged to the Ministry of Health [7].

As observed in previous national and international studies [6, 8, 10–12], arterial hypertension predominates in men and is the most frequently reported cardiovascular risk factor. Furthermore, as in the PERSTEMI I study [7] and in the Trial of Routine Angioplasty and Stenting after Fibrinolysis to Enhance Reperfusion in Acute Myocardial Infarction (TRANSFER-AMI) [12] study, anterior localization and Killip–Kimball I stage were the main forms of presentation in the present study. The mean age of the patients who underwent PPCI was significantly higher than those undergoing the PIs, which could be related to the decision to avoid the use of fibrinolytics in older patients due to increased risk of bleeding and, in turn, favoring the use of PPCI in older patients [13]. However, there were only 2 cases of major bleeding, both in the PPCI group, not related to second coronary procedures, because despite having multiarterial lesions the patients did not undergo another intervention, and thus, this was not considered the cause of bleeding. In one patient the femoral access was complicated with retroperitoneal bleeding, while the second case was by radial access and there was subarachnoid bleeding.

The time from symptom onset to FMC and the time to reperfusion were significantly shorter in the PIs group. This finding is consistent with the results of the French registry of Acute ST-elevation and non-ST-elevation Myocardial Infarction (FAST-MI) [14] that included 1714 patients, wherein the median time to reperfusion was 130 min for the PIs group and 300 min for the PPCI group, with no differences in mortality after 1–5 years of follow-up [15]. In the Korean Acute Myocardial Infarction registry (KAMIR) [16], the time from symptom onset to reperfusion was shorter in the PIs compared with the PPCI (165 vs. 241 min, *p* < 0.001) as was the time from the FMC to reperfusion therapy (80 vs. 145 min, *p* < 0.001) with no differences in mortality between groups.

Other randomized and observational studies reported total ischemic times ranging from 100 to 165 min for PIs and from 178 to 255 min for PPCI [3–5]. The Pharmacoinvasive Strategy vs. Primary PCI in STEMI: A Prospective Registry in a Large Geographical Area (PHASE-MX) [1] study conducted in Mexico reported a total ischemic time of 325 min for PIs and 320 min for PPCI. This is a remarkably smaller difference when compared with the findings obtained in our study for the PPCI (450 min).

In our study, the time from the FMC to reperfusion was 90 min for the PIs group and 287 min for the PPCI group. These are longer times than recommended by international guideline objectives [2, 17], which may be explained by failures in timely diagnosis, the lack of access to fibrinolytic agents, the need to refer patients to centers with greater response capacity, traffic conditions or geography, the lack of a unified ambulance system, health system fragmentation, emergency services overload, and the scarcity of centers with a catheterization room in Peru (there is only one hospital in Lima that can perform primary angioplasty 24/7). All these limitations suggest and support the hypothesis that the PIs could be considered the most timely and feasible option for the majority of patients in Peru.

The PIs group presented greater IRA patency in the baseline angiography, indicating that a greater number of patients in this group were transferred to the destination hospital with open arteries, which could lead (assuming that there more myocardium was previously rescued) to clinical benefits during follow-up. In addition, higher success rates (TIMI 3 flow) after angioplasty were achieved in the PIs group, which could have an impact on the reduction of mortality since previous studies had already reported that the degree of TIMI coronary flow after the procedure is independently correlated with mortality within one year after myocardial infarction [18]. These findings are similar to those of the Strategic Reperfusion Early After Myocardial Infarction (STREAM) [19] study wherein the final TIMI 3 flow was lower in patients who underwent PPCI and rescue PCI than in patients who had a scheduled angiography (within 6–24 h). Additionally, these findings are similar to those of a study conducted during the pandemic in China [20] reporting that patients who underwent fibrinolysis combined with delayed angioplasty (within the first 24 h) presented better TIMI flow after the procedure than those who underwent PPCI, with a similar rate of adverse in-hospital outcomes. Despite this evidence, a recent meta-analysis of randomized and observational studies [21] found no significant differences in final TIMI 3 flow between the PIs and the PPCI.

Like us, previous studies [4, 5, 15, 22] have described a similar safety and efficacy of the PIs and PPCI in reducing mortality and morbidity. Bainey et al. [3] observed better results with PIs and similar rates of major bleeding and intracranial hemorrhage in their study in Canada. These reports indicate that the PIs is an appropriate and reasonable alternative, with similar results to PPCI especially for Latin-American countries in which access to PPCI is limited.

An interesting finding in the group of patients who received fibrinolysis was a case of ischemic cerebrovascular event not related to atrial fibrillation, this is a very rare complication with a not well clarified pathophysiology, although several mechanisms have been proposed [23]. A large registry indicated that the incidence of in-hospital ischemic stroke after STEMI is about 1.2%, increasing the risk of mortality, and the factors associated include: age, female gender, atrial fibrillation, history of cerebrovascular accident, heart failure, diabetes mellitus, chronic renal failure, carotid artery disease, aortic disease, left ventricle thrombus, left ventricle aneurysm, acute or chronic deep venous thrombosis/pulmonary embolism, hypercoagulable state, and coronary artery bypass grafting [24].

In our study, the two strategies showed no differences in-hospital mortality, cardiovascular mortality, and postinfarction heart failure. However, in a similar study conducted in Mexico, Araiza-Garaygordobil et al. [1] found a trend towards a reduction in cardiovascular events with the use of the PIs. This leads to the conclusion that the greater the delay in establishing PPCI reperfusion, the worse the outcome [2, 25]. Other studies even observed that fibrinolysis was associated with a reduced mortality at one year in early presenters [26]. Additionally, a meta-analysis of patients in hospitals with no access to PPCI found that performing PIs reperfusion significantly reduced the short-term mortality when compared to receiving PPCI with 200 min or more between symptom onset to reperfusion [21]. In this report, even though the PIs group had a younger age, a shorter total ischemia time, and better TIMI flow after PCI, there were no significant differences in hospital outcomes, which may be due to the small sample size and could vary in favor of the PIs in the medium- or long-term follow-up or in higher-risk subgroups.

Our results are important for low- to middle-income countries such as Peru because they demonstrate that, regardless of the health system, in the PIs group the lytic treatment and the associated medical therapy with immediate or rapid routine transfer for PCI (rescue or systematic) are beneficial and necessary and improve clinical results. In previous real-world studies such as the CARESS in AMI study [27] (in which half doses of reteplase and abciximab were used in high-risk patients), similar results were found even with the use of different schemes. Therefore, our study supports the need to place greater emphasis on improving fibrinolysis and transfer network systems in low- to middle-income countries without widespread availability of timely PPCI. In addition, in registries such as EUROTRANSFER [28], it has been reported that among the group of patients transferred for PPCI, only a minority (36%) were treated within the recommended 90-min time window. Therefore, in countries such as Peru, the PIs should be considered the best option.

Some limitations affect the external value of our study. The PERSTEMI registry was an observational study wherein most of the patients enrolled belonged to the social security health system in the city of Lima, and were finally treated in a national reference institute. Therefore, the findings may not reflect the situation of the entire Peruvian population. In addition, the small sample size of this substudy may limit its ability to detect statistically significant differences between the groups studied to be extrapolated to the population. The fact that the registry was elaborated during 2020 in the midst of the COVID-19 pandemic may have led to longer ischemic times in the PPCI group and also reduced the number of cases from all over the country.

## Conclusions

In this real-world study, the in-hospital mortality and heart failure rates did not differ among patients with STEMI who underwent the PIs or PPCI approach. The PIs was performed within a shorter time and achieved more successful coronary flow of the IRA post-PCI compared to PPCI. These findings suggest that fibrinolysis performed with a PIs is a feasible, effective, safe, and time-effective alternative in low- and middle-income countries like Peru. Therefore, it should be promoted as the first-choice intervention in most public health care systems in Peru to improve clinical outcomes, especially in the absence of established primary angioplasty programs.

## Data Availability

The datasets used and/or analyzed during the current study are available from the corresponding author on reasonable request.

## Abbreviations

PPCI: Primary percutaneous coronary intervention
PCI: Percutaneous coronary intervention
IRA: Infarct-related artery
IQR: Interquartile range
CI: Confidence interval
RR: Relative risk
TIMI: Thrombolysis in myocardial infarction
STEMI: ST-segment elevation myocardial infarction
PIs: Pharmacoinvasive strategy
PERSTEMI II: Second Peruvian Registry of STEMI
PERSTEMI I: First Peruvian Registry of STEMI
h: Hour
FMC: First medical contact
